# Ensemble of LinkNet Networks for Head and Neck Tumor Segmentation

**DOI:** 10.1007/978-3-031-83274-1_16

**Published:** 2025-03-03

**Authors:** Maria Baldeon-Calisto

**Affiliations:** Departamento de Ingeniería Industrial and Instituto de Innovación en Productividad y Logística CATENA-USFQ, Universidad San Francisco de Quito USFQ, Quito, Ecuador

**Keywords:** Head and Neck Cancer, Tumor Segmentation, Convolutional Neural Networks, Deep Learning

## Abstract

The segmentation of head and neck cancer (HNC) tumors is a critical step in radiotherapy treatment planning. The development of automatic segmentation algorithms has the potential to streamline the radiation oncology process. In this work, we develop an ensemble of LinkNet networks for HNC tumor segmentation as part of the HNTS-MRG 2024 Grand Challenge. A single LinkNet network, pretrained on the Imagenet dataset, was trained for 200 epochs on the HNC dataset provided by the challenge. Eight good performing weights from the internal validation set were selected to create an ensemble of 2D networks. Specifically, each selected weight was used to generate a LinkNet architecture, resulting in eight networks whose predictions were averaged to produce the final predicted segmentation. Our experiments demonstrate that the ensemble network performs better than each individual architecture, leveraging the benefits of ensemble learning without the computational cost of training each network from scratch. In the challenge’s test set, the LinkNet Ensemble (team ECU) achieved an aggregated Dice score of 64.60% and 49.53% for metastatic lymph nodes and primary gross tumor segmentation, respectively, and a mean score of 57.06%.

## 1 Introduction

Head and neck cancers (HNC) comprise a diverse group of malignancies that affect the upper aerodigestive tract. Among these, squamous cell carcinoma is the most prevalent type [1], with most cases occurring in the oral cavity, oropharynx, and larynx. Globally, HNC accounts for approximately 5% of all cancers, with an annual incidence of 600,000 new cases and 300,000 related deaths [2]. Major risk factors include alcohol consumption, infections by human papilloma virus and Epstein-Barr virus, and tobacco use. Despite advances in treatment, the prognosis for patients remains poor, with a 5-year survival rate of approximately 45.7% [3]. Existing treatment modalities, such as surgery, radiotherapy, and targeted therapies, face significant challenges, including late diagnosis, metastasis, and therapy resistance. Moreover, most therapies demonstrate higher efficacy in early-stage disease.

Accurate segmentation of medical images is critical in radiotherapy treatment planning, as it allows for precise delineation of tumors and surrounding healthy tissues. This is essential for delivering targeted radiation doses while minimizing exposure to nearby organs. However, manual segmentation, which requires radiation oncologists to manually outline regions of interest on imaging scans, is not only time-consuming and labor-intensive but also subject to inter-observer variability. Hence, there is a pressing need for the development of automated segmentation methods, which can significantly reduce time and cost involved while improving consistency.

Recent advances in deep learning have shown promising results in the automatic segmentation of head and neck organs-at-risk (OARs). In [4], a fully convolutional neural network combined with a shape representation model is proposed. The network achieved high levels of accuracy across multiple OARs, surpassing the performance of atlas-based methods. Similarly, Nikolov et al. [5] presented a 3D U-Net architecture that provided segmentation results comparable to expert-level performance in the delineation of various head and neck OARs. Jinzhong et al. [6] proposed a multichannel Gaussian mixture model algorithm that integrates data from CT, PET, and MRI scans for tumor volume delineation. The model also showed to have a strong concordance with physician-defined gross tumor volumes. Despite these advancements, automatic segmentation techniques are not yet sufficiently robust to fully replace physician-drawn volumes, particularly in the delineation of gross tumor volumes.

In this work, we propose an ensemble of LinkNet models for the segmentation of metastatic lymph nodes and primary gross tumor in T2-weighted magnetic resonance images. A LinkNet architecture [7] was trained on a head and neck tumor dataset provided by the HNTS-MRG 2024 Grand Challenge. Based on internal validation, eight well-performing model weights were selected to create an ensemble of LinkNet networks. The final predicted segmentation was generated by averaging the predictions from these eight networks. Our experiments show that the ensemble approach surpasses individual network performance in segmenting metastatic lymph nodes (*GTV_n_*) and primary gross tumors (*GTV_p_*). On the challenge test set, the LinkNet ensemble achieved an aggregated Dice score of 64.60% for *GTV_n_* segmentation and 49.53% for *GTV_p_* segmentation.

### 2 Methods

### 2.1 Imaging Data

The dataset used in this work was sourced from the HNTS-MRG 2024 Grand Challenge. It is comprised of 150 training and 50 test cases, all from patients with histologically confirmed HNC who received radiotherapy at the University of Texas MD Anderson Cancer Center. The dataset includes T2-weighted magnetic resonance anatomical sequences of the head and neck region with a mix of fat-suppressed and non-fat suppressed sequences. The images have been annotated by 3 to 4 experts that delineate the *GTV_p_* and *GTV_n_* and the concesus segmentation are made available to the participants.

### 2.2 Image Preprocessing

The preprocessing pipeline was designed to standardize image resolution and intensity values across the dataset. First, all images were resampled to an isotropic resolution of 0.5 × 0.5 × 2 mm using the B-Spline interpolation method for the images and nearest neighbor interpolation for the ground truth segmentation masks. Next, pixel intensities were normalized by truncating values that fell outside three standard deviations from the mean intensity. Any pixel intensities beyond this threshold were clipped to the respective 3-standard deviation limits. Lastly, intensities were rescaled to a range between 0 and 1 by subtracting the minimum intensity value and dividing by the range.

The dataset, consisting of 150 cases, was split into training and validation sets with an 80–20 ratio. This resulted in 120 cases for training, totaling 8,808 slices, and 30 cases for validation, consisting of 2,186 slices.

### 2.3 Model Architecture

**Fig. 1.**
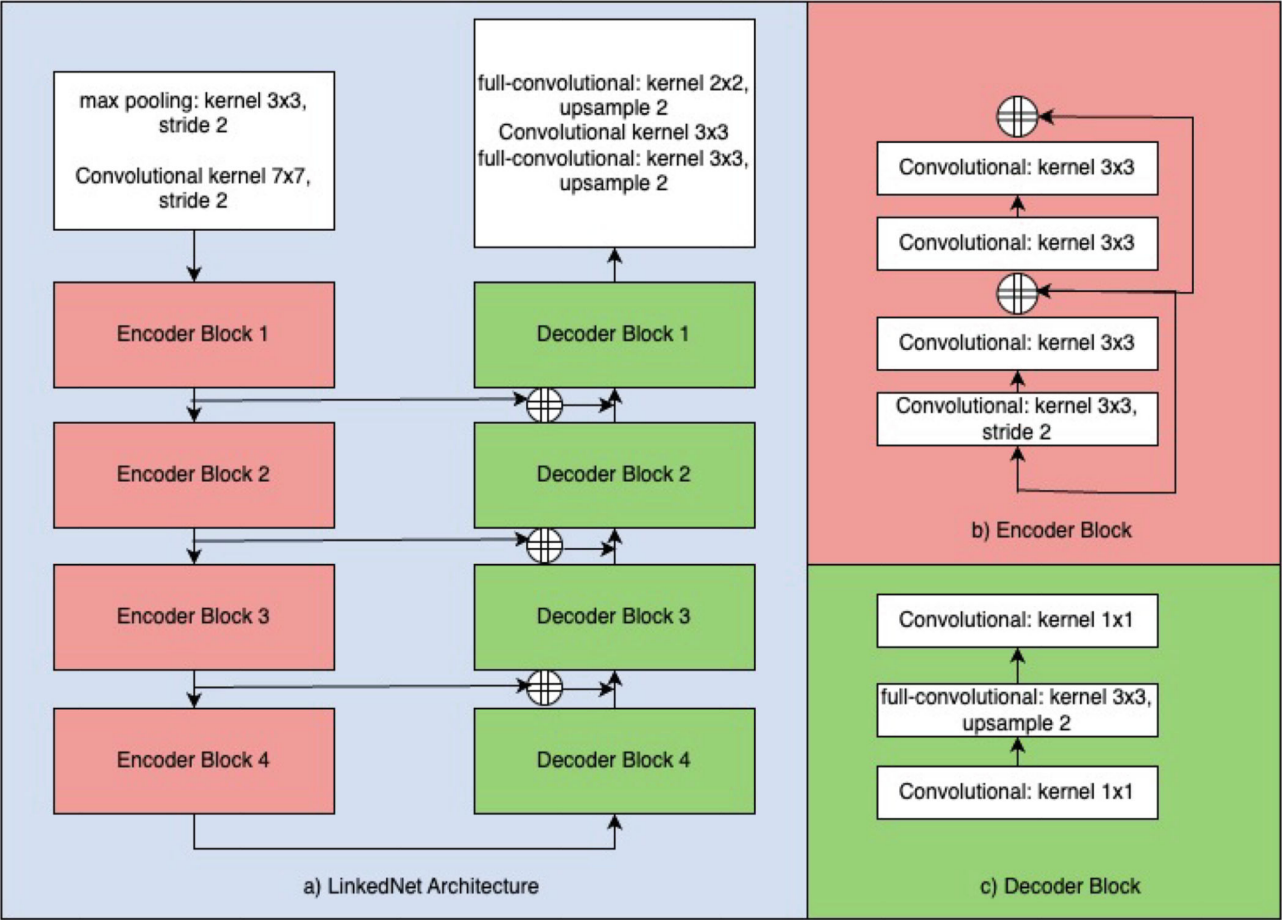
LinkNet architecture [7] comprised of an up-sampling and down-sampling path.

We employed an ensemble of LinkNet architectures [7], as this model has demonstrated strong performance in various segmentation tasks and offers computational efficiency [8,9]. LinkNet is a fully-convolutional network featuring a down-sampling and up-sampling path, as ilustrated in Fig. 1a). The down-sampling path consists of an initial block followed by 4 encoder blocks, while the up-sampling path comprises 4 decoder blocks and a final block. The initial block applies a convolutional layer with a 7 × 7 kernel and a stride of 2, followed by spatial max-pooling with a 3 × 3 kernel and a stride of 2. Convolutional layers have a ReLU activation function, with batch normalization applied between layers. Each encoder block contains two residual blocks, as presented in Fig. 1b). The decoder blocks in the up-sampling path are composed of three convolutional layers, depicted in Fig. 1c). The final block includes an up-sampling full convolutional layer with a 3 × 3 kernel size, a convolutional layer of the same kernel size, and a final full convolutional layer. The LinkNet model was imported from pytorch’s segmentation models library. As LinkNet is a 2D architecture, the input to the model were the 2D slices from the 3D pre-RT images.

### 2.4 Training Configuration

The LinkNet model was initialized with pre-trained weights from the Imagenet dataset and fine-tuned in the HNC dataset for 200 epochs, stopping once the validation Dice score showed no further improvement. We utilized an Adam optimizer with a learning rate of 2 × 10^−3^ and a batch size of 20 slices. Moreover, the model was trained with a dice loss function to optimize performance. During training, data augmentation on-the-fly was applied to increase the size and diversity of the dataset using the albumentations library. Specifically, horizontal and vertical flips were applied with a probability of 0.5, along with rotations and transpositions. The implementation use Python 3.8.18, Pytorch 2.4.0 and executed on a Nvidia Tesla V100 GPU.

To construct the ensemble, we selected eight weights that exhibited both high validation and training Dice scores. To ensure diversity among the selected models, a minimum difference of at least eight epochs between them was enforced. Furthermore, we verified that the models had different performance in the validation cases and hence made distinct mistakes. This approach proved effective, as the ensemble performed better than individual models, demonstrating the benefits of combining multiple networks with complementary strengths.

## 3 Results and Discussion

The performance of the eight ensemble members, referred to as LinkNet, and the eight-network ensemble, called Ensemble_8, on the internal validation set in terms of mean Dice score, is presented in Table 1. Additionally, we evaluated a ten-network ensemble by selecting 10 weights from the training process, denoted as Ensemble_10. We use the conventional Dice score, that assigns a value of 0 for a false positive prediction, and a dice of 1 for an true negative prediction. As shown, Ensemble_8 and Ensemble_10 exhibit nearly identical performance, both achieving higher segmentation accuracy than any individual ensemble member. Furthermore, the results suggests that increasing the number of networks beyond eight does not substantially improve accuracy, indicating a point of diminishing returns.

**Fig. 2.**
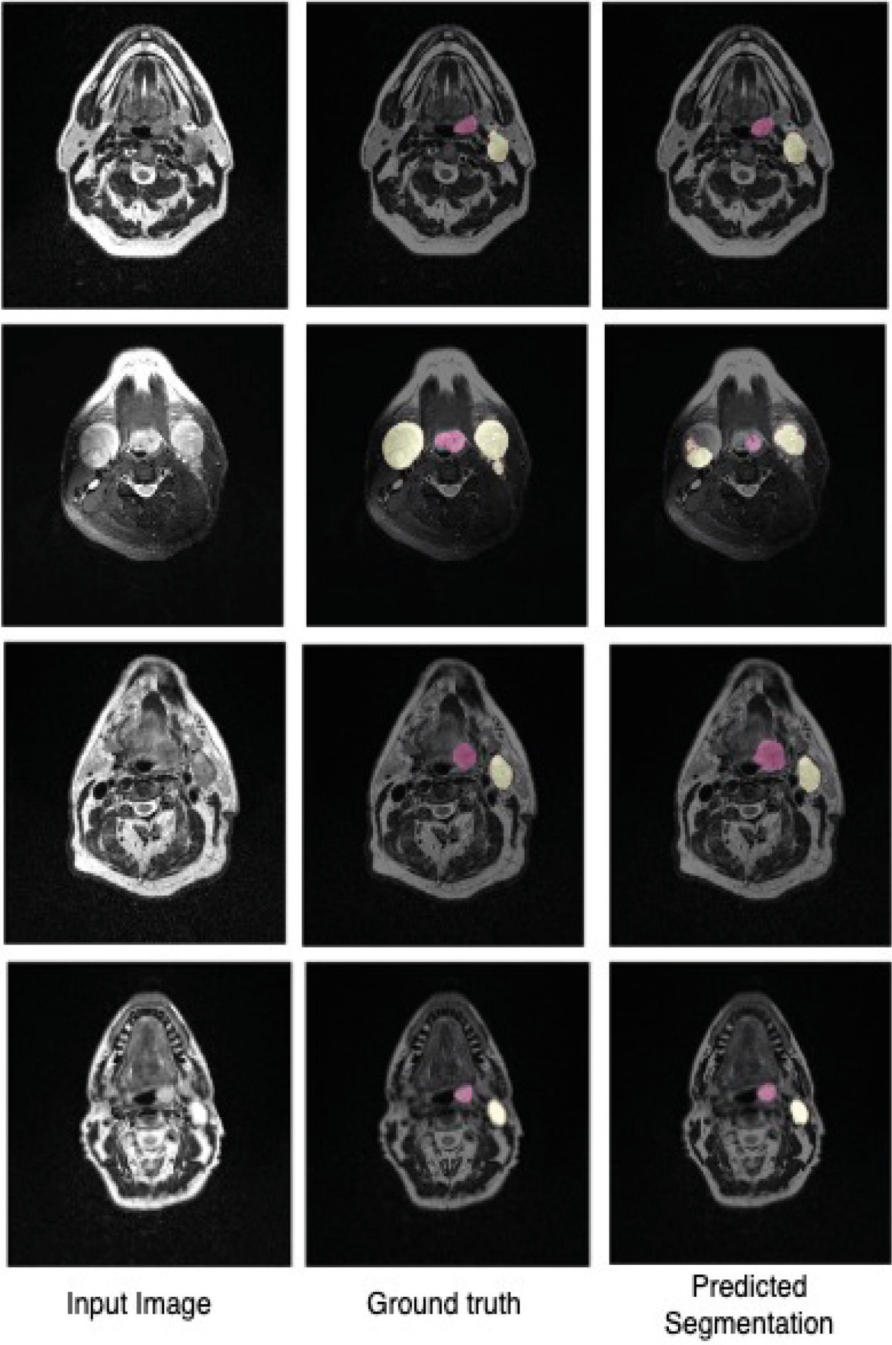
Examples of segmentations generated by the ensemble of LinkNet networks. The red region is the primary gross tumor and the yellow region the metastatic lymph nodes.

Paired t-tests were conducted to assess the significance of the difference between Ensemble_8 and each LinkNet network. At a 95% confidence level, the difference between the ensemble and each network was found to be statistically significant, allowing us to conclude that the ensemble network achieves the highest mean score in segmenting metastatic lymph nodes and primary gross tumors. This finding highlights the advantages of ensemble learning, as combining predictions from multiple models results in a more robust and accurate segmentation, demonstrating the potential of this approach in clinical applications where precise tumor delineation is critical for effective treatment planning. Qualitative examples of the segmentation can be seen in Fig. 2.

For external validation, we submited our model to the challenge’s website through a docker container. The LinkNet ensemble network achieves a mean aggregated Dice score of 64.60% and 49.53% for *GTV_n_* and *GTV_p_* segmentation, respectively.

The limitations of our work include the use of a 2D network for the segmentation of 3D images, a choice driven by time and computational constraints. Additionally, as the ensemble was created from a single LinkNet network, there is a degree of correlation between the performance of the ensemble members. Incorporating models initialized with different weights or using distinct architectures could potentially improve performance. However, our statistical results demonstrate that integrating the networks through an ensemble does improve segmentation accuracy for both types of tumors. This suggests that our approach could be a viable strategy in situations where computational resources and time are limited. Additionally, while our study focused exclusively on Task 1 of the challenge (pre-RT segmentation), extending our method to mid-RT segmentation prediction (Task 2) could be a valuable direction for future research, potentially demonstrating the model’s adaptability to dynamic treatment scenarios.

## 4 Conclusions

In this work, we developed an ensemble of LinkNet networks for head and neck cancer (HNC) tumor segmentation. By leveraging a single LinkNet network pretrained on the ImageNet dataset, we trained the model for 200 epochs on the HNC dataset provided by the HNTS-MRG 2024 Grand Challenge. From the internal validation set, we selected eight high-performing weights to create an ensemble, resulting in improved segmentation accuracy compared to individual networks. This ensemble approach offers the advantages of ensemble learning without the added computational cost of training each network independently. In the challenge’s test set, the LinkNet ensemble achieved aggregated Dice scores of 64.60% and 49.53% for metastatic lymph nodes and primary gross tumor segmentation, respectively.

## Figures and Tables

**Table 1.** Ablation Studies on the internal validation set. Mean dice values are presented.

Model	Dice *GTV_p_*	Dice *GTV_n_*
LinkNet_1	0.769	0.825
LinkNet_2	0.789	0.818
LinkNet_3	0.781	0.824
LinkNet_4	0.798	0.807
LinkNet_5	0.791	0.818
LinkNet_6	0.795	0.809
LinkNet_7	0.800	0.812
LinkNet_8	0.794	0.811
Ensemble_8	0.822	0.845
Ensemble_10	0.821	0.846
